# USING THE INSTITUTE OF HEALTHCARE IMPROVEMENT'S 4M FRAMEWORK TO IMPROVE DISASTER PREPAREDNESS FOR NH POPULATIONS

**DOI:** 10.1093/geroni/igac059.1381

**Published:** 2022-12-20

**Authors:** David Dosa, Dylan Jester, Lindsay Peterson, Debra Dobbs, Lisa Brown, Kathy Black

**Affiliations:** Brown University, Providence, Rhode Island, United States; University of California San Diego, La Jolla, California, United States; University of South Florida, Tampa, Florida, United States; University of South Florida, Tampa, Florida, United States; Palo Alto University, Palo Alto, California, United States; University of South Florida, Sarasota-Manatee, Florida, United States

## Abstract

Prior studies have repeatedly demonstrated that exposure to natural disasters such as hurricanes, earthquakes, and flooding events have profound effects on nursing home (NH) residents. Previous efforts to improve disaster preparedness have often been predicated on the decision to evacuate or shelter in place from a disaster. Unfortunately, evacuation of NH residents is often not possible and nursing homes are often required to make difficult decisions to protect the lives of their residents without data. Recognizing that healthcare systems increasingly provide care for complex individuals; the Institute of Healthcare Improvement (IHI) and the John H. Hartford Foundation introduced a framework for evaluating age-friendly healthcare systems based on 4 evidence-based core elements that matter to older adults. We believe that this 4 M’s paradigm: What Matters, Medication, Mentation, and Mobility, provides a step off point for defining a practical approach to disaster preparedness for nursing homes exposed to future disasters.

